# Molecular neuropathology: an essential and evolving toolbox for the diagnosis and clinical management of central nervous system tumors

**DOI:** 10.1007/s00428-023-03632-4

**Published:** 2023-09-02

**Authors:** Luca Bertero, Luca Mangherini, Alessia Andrea Ricci, Paola Cassoni, Felix Sahm

**Affiliations:** 1 Pathology Unit, Department of Medical Sciences, University of Turin and Città Della Salute E Della Scienza University Hospital, Via Santena 7, 10126 Turin, Italy; 2 Department of Neuropathology, Heidelberg University Hospital, Im Neuenheimer Feld 672, 69120 Heidelberg, Germany; 3 Clinical Cooperation Unit Neuropathology, German Cancer Research Center (DKFZ), German Consortium for Translational Cancer Research (DKTK), Heidelberg, Germany

**Keywords:** Neuropathology, Molecular pathology, DNA methylation profiling, NGS, CNS tumors

## Abstract

Molecular profiling has transformed the diagnostic workflow of CNS tumors during the last years. The latest WHO classification of CNS tumors (5th edition), published in 2021, pushed forward the integration between histopathological features and molecular hallmarks to achieve reproducible and clinically relevant diagnoses. To address these demands, pathologists have to appropriately deal with multiple molecular assays mainly including DNA methylation profiling and DNA/RNA next generation sequencing. Tumor classification by DNA methylation profiling is now a critical tool for many diagnostic tasks in neuropathology including the assessment of complex cases, to evaluate novel tumor types and to perform tumor subgrouping in hetereogenous entities like medulloblastoma or ependymoma. DNA/RNA NGS allow the detection of multiple molecular alterations including single nucleotide variations, small insertions/deletions (InDel), and gene fusions. These molecular markers can provide key insights for diagnosis, for example, if a tumor-specific mutation is detected, but also for treatment since targeted therapies are progressively entering the clinical practice. In the present review, a brief, but comprehensive overview of these tools will be provided, discussing their technical specifications, diagnostic value, and potential limitations. Moreover, the importance of molecular profiling will be shown in a representative series of CNS neoplasms including both the most frequent tumor types and other selected entities for which molecular characterization plays a critical role.

## Introduction

The evaluation of specific molecular hallmarks has become a mandatory step for diagnosing central nervous system (CNS) tumors since multiple years. Thanks to the publication of the 4th revised edition of the World Health Organization (WHO) classification of CNS tumors in 2016 [1], the concept of an integrated histopathological and molecular diagnosis has become a cornerstone of oncological neuropathology.

The integrated diagnosis is a diagnosis based on multiple layers which, starting from the conventional histopathological features, is enriched by molecular information. Molecular profiling can help or even provide by itself the diagnostic classification of a tumor type and/or improve the prognostic stratification by complementing histology-based tumor grading and/or help tailor treatment by disclosing potential therapeutic targets. These layers are frequently intertwined and a solid background knowledge about the interpretation of each molecular marker in the relevant diagnostic context is required for their correct translation into diagnostic practice and clinical management.

The latest 2021 WHO classification of CNS tumors (5th edition) [2] has further expanded the significance of molecular profiling in this setting and to date, all the most frequent CNS tumor types envisage the evaluation of a subset of molecular markers in their diagnostic workflow. Additionally, many novel tumor types were only discovered through these technologies, e.g., DNA methylation analysis.

**Fig. 1:**
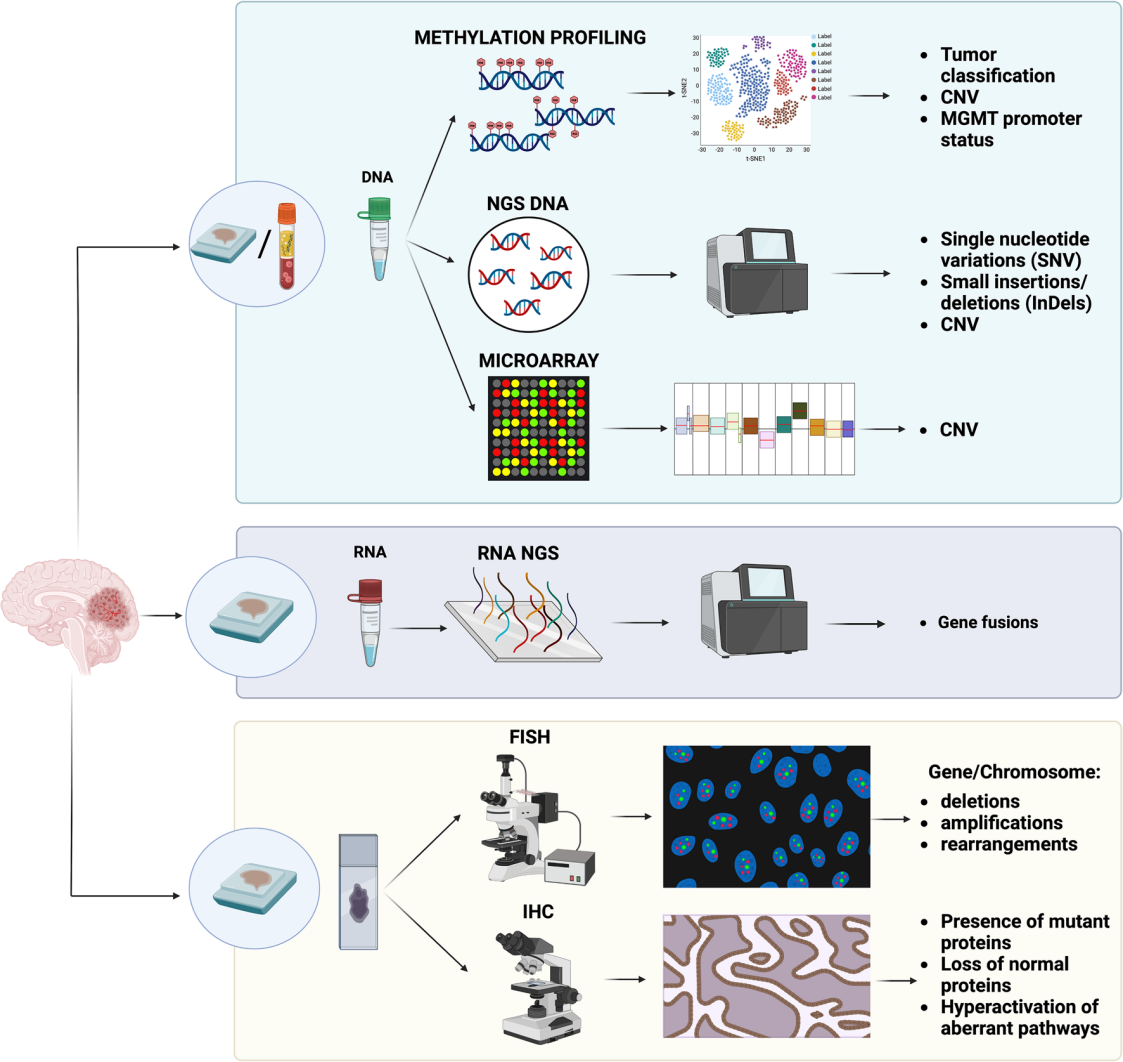
Graphical overview of the main current molecular assays which are relevant for molecular neuropathology

From a more technical point of view, molecular neuropathology now encompasses a wide and quickly evolving range of assays with varying aims, complexity, availability, and costs (Fig. 1) [3]. Being aware of these specifications is important since it helps to choose the most appropriate tool for the diagnostic problem that is being tackled and to correctly interpret the results.

The aim of this review is thus twofold: (i) to provide a brief, but comprehensive outlook of the available molecular tools and assays which are currently the most relevant for CNS tumor diagnosis; (ii) to provide an overview of the key molecular markers required for the assessment of a representative series of CNS neoplasms.

## Molecular assays relevant for diagnosis and treatment of CNS tumors

### DNA methylation profiling

DNA methylation profiling has arguably been the most impactful molecular tool in the recent years concerning the diagnostic classification of brain tumors [4]. In brief, this approach exploits the capability of tumor cell epigenetic profile to recapitulate both the characteristics of the tissue of origin and the changes acquired during the oncogenic transformation, thus providing a specific signature for each tumor type. Moreover, additional valuable information can be derived regarding tumor copy number variations (CNV) which can help to further confirm the diagnosis and/or provide additional molecular stratification [5].

Practically, DNA methylation profile is currently assessed using the MethylationEPIC array beadchip (850K) allowing to investigate the methylation status of several hundred thousand CpG islands across the whole genome. The raw output data (IDAT files) is then uploaded into a dedicated platform (https://www.molecularneuropathology.org/) and matched with a representative repository of CNS tumors and other selected entities. A matching score (≥ 0.9) strongly supports the achieved diagnostic classification, although it should be noted that the results should always be reviewed by expert neuropathologists in the context of the clinical, imaging, and histopathological findings as well as of other identified molecular traits. However, it should be noted that DNA methylation profiling using this platform is not a certified assay and thus it must not be directly used for diagnostic procedures, but, depending on local regulations, a pipeline including this analysis may be set-up as a laboratory-developed test.

Moreover, DNA methylation profiling often allows tumor subgrouping into clinically relevant entities. For example, medulloblastomas can be divided into more than ten subgroups with significantly different clinical features and outcomes [6].

This approach has been very fruitful both for research and daily diagnostic purposes. Concerning the first aim, many novel tumor types and subtypes have been discovered or comprehensively characterized thanks to the unsupervised analysis of large datasets of brain tumors [7–12]. Many of the newly identified tumors show significant overlaps with other entities in terms of morphological features and/or a very low incidence, contributing to explain why they were previously unrecognized as distinct neoplasms by conventional light microscopy.

Concerning the implementation into the daily diagnostic workflow, multiple groups have now published their experience both in pediatric and adult settings [13–16]. Overall, a matching (≥ 0.9) score is achieved in about 50–65% of samples and a significant impact on the diagnosis has been observed in about 10–20% of cases with potential clinical consequences. This is a remarkable finding justifying the quickly acquired practical relevance of this tool in so few years. Many interesting insights can be gathered by analyzing these results. Higher median classification scores are usually observed in cases being analyzed to confirm a diagnosis or to assess the specific tumor subtype; conversely, a wider range of scores is observed among challenging samples or smaller specimens [13]. The reasons why lower scores can be obtained in cases deemed complex following the initial histological examination are multiple. A first explanation can lie in sample characteristics: for example, a small, poor quality, and/or unrepresentative biopsy can be unclassifiable both for the pathologist and through methylation profiling. This consideration highlights the importance of the quantity and quality of the submitted DNA for analysis. Another possibility is that the submitted neoplasm is still unrecognized according to the current diagnostic criteria and under-represented in the used DNA methylation classifier version. This occurrence was more frequent with the initial versions of the classifier since many novel entities were then identified thanks to their specific methylation profile.

Ideally, 200 ng of DNA with ≥ 60% tumor cell concentration is desirable, even though a diagnostic classification can be achieved with significantly lower amounts. In terms of sample type, formalin fixed paraffin embedded (FFPE) tissue blocks are usually used with similar results compared to fresh frozen samples and analysis of older specimen can also result in correct classification [16]. DNA methylation profiling has also been shown to be particularly useful to reclassify rare tumor types with unspecific histopathological characteristics [17].

Nowadays, DNA methylation profiling is surely a key tool for diagnosing CNS tumors even though this approach poses significant challenges in terms of the required technological facilities, costs, turnaround time (multiple days), and the required expertise for its execution and correct interpretation.

In terms of future outlooks, it should be noted that both the specific assay used for DNA methylation profiling and the classifier tool are subject to longitudinal changes. For instance, a novel version of the MethylationEPIC beadchip (v2.0) has been recently introduced and novel versions of the brain tumor classifier have been developed since the initial 2018 classifier [4] to account for the newly defined tumor groups. These variations should be taken into consideration both when implementing DNA methylation profiling and when re-evaluating classification results over time.

### DNA and RNA sequencing

Many CNS tumors are characterized by specific point mutations which can be identified by DNA sequencing or gene fusions which can be mainly detected by RNA sequencing. For example, mutations in *IDH1*/*IDH2* and H3-coding genes characterize specific subsets of adult and pediatric gliomas, respectively [18, 19]. Other mutations, like BRAF V600E, are promiscuous and can be present, with varying frequencies, in multiple tumor types, but can anyway contribute to the diagnostic assessment and enable a targeted treatment [20].

Many assay types can be used for DNA sequencing including single gene direct sequencing (i.e., Sanger sequencing) and next generation sequencing (NGS)-based approaches like targeted panel sequencing and whole exome/genome sequencing (WES/WGS). In general, these analyses allow to detect single nucleotide variations (SNV), small insertions/deletions (InDel), and, based on the overall extension of the targeted regions, CNV. Gene fusions can also be detected, but with limited efficacy since most of these alterations involve non-coding regions which are not or partially represented in most NGS assays [21].

Within NGS assays, targeted gene panel sequencing is the most relevant tool to date for the daily molecular diagnostic work up of CNS tumors since it allows the analysis of relatively large sets of relevant genes with acceptable costs, turnaround times, and interpretation feasibility. Of note, many of the genes which are mostly relevant for the diagnosis of brain tumors are relatively specific to these neoplasms; thus, use of customized or larger panels is often warranted. The diagnostic efficacy of medium-sized gene panels has been demonstrated, allowing to detect mutations and CNV even with limited input material [22]. These results were confirmed by more recent studies employing larger gene panels (e.g., *IDH1*/*IDH2*, *TERT*, *TP53*, *ATRX*, *BRAF*, *H3F3A*, *H3F3B*) [23–26]. By using these assays, diagnostically relevant alterations can be detected in more than half of the analyzed CNS tumors. Moreover, Ji J et al. reported that informative CNV were detected even in 57% of cases with noncontributory NGS results.

Laboratory protocols are critical in this setting as discussed for DNA methylation profiling. DNA quality and tumor cell rate should be maximized and adequate coverage/read depth should be achieved according to the assay type and sample characteristics [27]. The data analysis pipeline and the expertise of the reporting molecular pathologist are also of utmost importance for correct variant calling and interpretation. Finally, detection of potential germline alterations is becoming more frequent due to larger panel sizes and it should be noted that management of these occurrences has to be tailored according to national/local guidelines and regulations.

Analysis of circulating tumor DNA (ctDNA) is another possibility to achieve tumor molecular profiling through minimally invasive blood and/or CSF analysis. Technical challenges have so far hindered the implementation of these liquid biopsy assays in the daily practice, but data has been recently reported regarding the use of comprehensive NGS panels even on ctDNA, allowing to also detect CNV and addressing intratumoral heterogeneity [28].

Concerning RNA sequencing, the main aim of this analysis for diagnostic purposes is to detect gene fusions and many CNS tumors are characterized by these alterations. For instance, pilocytic astrocytomas frequently harbor the *KIAA1549*::*BRAF* fusion and specific molecular supratentorial ependymoma subtypes are defined by the presence of *ZFTA* or *YAP1* fusions [2]. Gene fusions are not only relevant for diagnosis, but can also represent exploitable therapeutic targets; for example, infant-type hemispheric gliomas are frequently characterized by *NTRK1*/*NTRK2*/*NTRK3*, *ROS1*, *ALK*, or *MET* gene fusions for which effective inhibitors are available.

Gene rearrangements can result in oncogenic activity by multiple mechanisms including acquisition of constitutive activity or by promoting the transcription of the resulting fusion gene [29, 30]. Many techniques can be employed for detecting gene fusions including FISH, RT-PCR, real-time RT-PCR, and RNA NGS, including targeted and whole transcriptome RNA NGS. RT-PCR, real-time RT-PCR, and targeted RNA NGS enable the analysis of a predetermined subset of genes with each assay, but do not allow to identify novel gene partners, a significant limitation for highly promiscuous genes like the *NTRK* family [31]. Nevertheless, targeted RNA NGS can be more effective with challenging samples and requires less complex bioinformatic pipelines, making it well suited for the routine diagnostic activity while whole transcriptome NGS enables the discovery of novel fusion partners, even if non-coding regions are involved. Concerning the potential technical pitfalls, RNA preservation in FFPE material is more limited compared to DNA; thus, analysis of older samples can more easily fail.

Studies focused on the significance of RNA NGS in CNS tumors have shown that this tool is especially worthy for pediatric neoplasms since they are more frequently characterized by these events like pilocytic astrocytoma, supratentorial ependymomas, *MYB*-/*MYBL1*-altered diffuse astrocytoma, angiocentric glioma, infant-type hemispheric glioma, and *MN1*-altered astroblastoma [32, 33]. In adult brain neoplasms, gene fusions are relatively rare and they usually do not represent a therapeutic target [34].

### Other tools

Microarray-based assessment of whole-genome CNV is another relevant diagnostic tool which has been frequently used to molecularly characterize CNS tumors, especially prior to the introduction of DNA methylation profiling. These assays allow to detect many chromosomal alterations (e.g., deletions, amplifications, loss of heterozygosity, copy-neutral loss of heterozygosity, chromothripsis,…) which are diagnostic and/or prognostic hallmarks of specific tumor types (e.g., 1p/19q codeletion, *EGFR* amplification, *CDKN2A/B* deletion…).

Nevertheless, molecular profiling does not necessarily mean the simultaneous analysis of multiple alterations. For example, FISH can be used to evaluate specific DNA loci directly on tissue slides and can be useful for validation purposes or if a specific alteration is strongly suspected based on the histopathological characteristics of a tumor or if the available material is insufficient for other types of analysis. FISH can be used to evaluate gene/chromosome deletions (e.g., 1p/19q codeletion), amplifications (e.g., *EGFR* amplification), and rearrangements (e.g., *KIAA1549*::*BRAF* in pilocytic astrocytoma) by using specifically designed probes.

Instead of nucleic acids, proteins can also be evaluated using widely available, fast, and inexpensive immunohistochemical stainings. Immunohistochemistry can be used to establish the presence of mutant proteins (i.e., IDH1 R132H, p53, H3 K27M, H3 G34R/V, BRAF V600E), the loss of normal/functioning proteins (ATRX, H3 K27me3, INI1, BRG1), or the hyperactivation of aberrant pathways (EZHIP). In addition to the low turnaround time, immunohistochemistry can be performed on very small biopsy samples and allows correlations with morphological features.

Finally, assessment of *MGMT* promotor methylation remains a mainstay of IDH-wildtype glioblastoma molecular characterization due to its prognostic and predictive relevance. Multiple assays can be used to investigate this marker and no one has shown a clear superiority in terms of clinical correlations. Since no equivalence criteria between the different assays are available, it is important to be aware of the specific characteristics of the locally available/selected assay type. It is also important to note that *MGMT* immunohistochemistry is not a reliable surrogate of these assays [35].

## Applying molecular assays to the diagnostic workup of CNS tumors: a series of representative examples

Molecular analyses can contribute or are required for the diagnosis of many of the tumor types envisaged by the 2021 WHO classification. The most relevant molecular tools vary according to the specific neoplasm and the following examples have been selected to show the significance of the previously discussed molecular tools for the current neuropathological practice.

### Adult diffuse gliomas

**Fig. 2:**
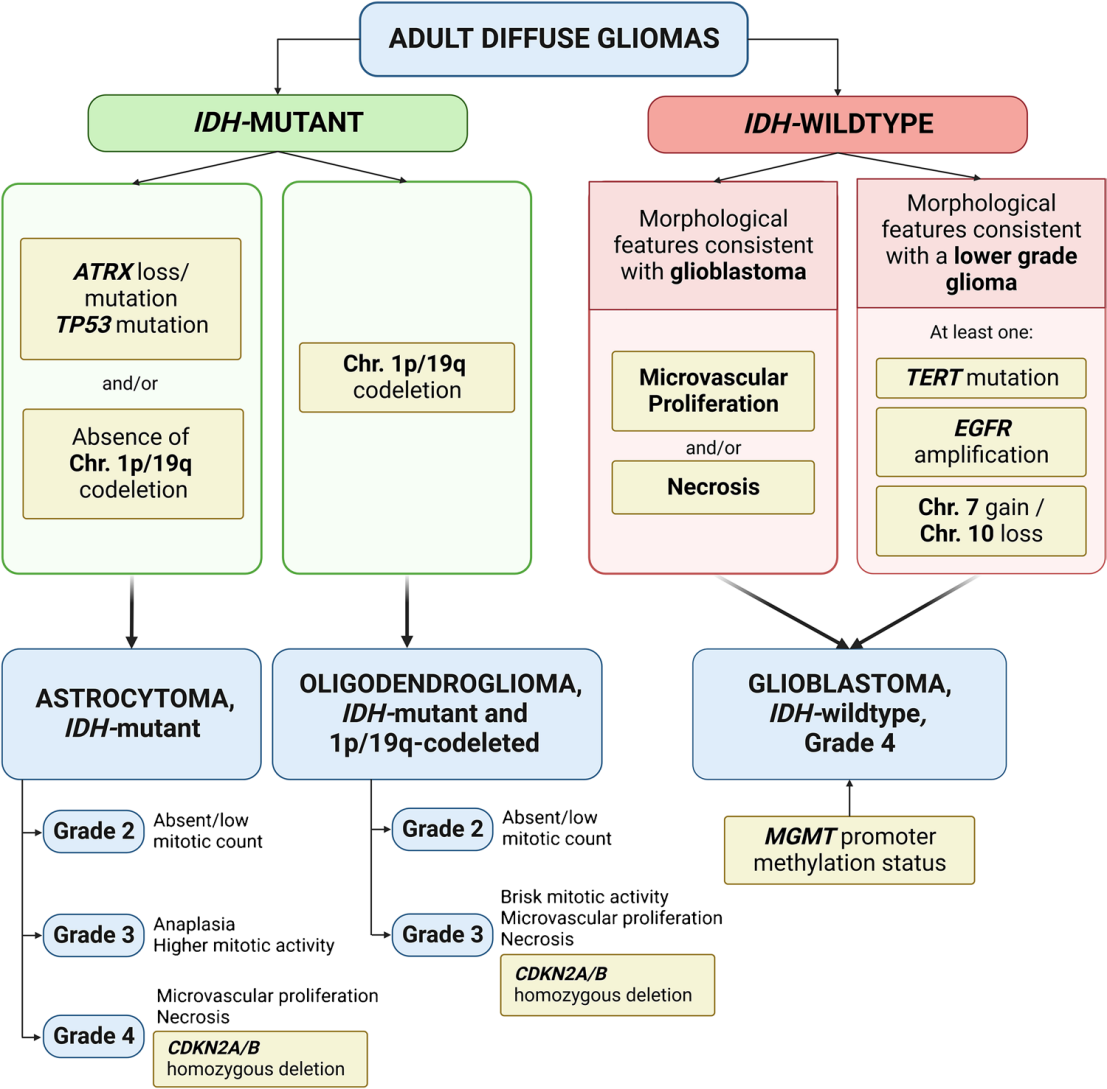
Diagnostic workflow of adult diffuse gliomas according to the latest WHO 2021 classification of central nervous system tumors showing the interplay between morphological and molecular markers for diagnostic classification and tumor grading

According to the 2021 WHO classification, adult diffuse gliomas are mainly stratified according to *IDH1*/*IDH2* status (Fig. 2). This division is well justified based on the different tumor biology, oncogenic mechanisms, and clinical implications according to this molecular marker [36].

Glioblastoma, IDH-wildtype is the most frequent diffuse glioma, usually occurring in older adults and harboring a dismal prognosis [37]. Glioblastoma is a morphologically and molecularly heterogenous neoplasm; histology usually shows a poorly differentiated astrocytic neoplasm with infiltrative growth, high proliferation, microvascular proliferation, and necrosis. Presence of at least one of the latter two criteria is necessary for the histopathological diagnosis of glioblastoma. In presence of a consistent morphology, it is necessary to exclude a *IDH1*/*IDH2* mutation and thus the diagnoses of an IDH-mutant astrocytoma or IDH-mutant, 1p/19q-codeleted oligodendroglioma [18].

If we look to our toolbox, multiple options are available for this task: immunohistochemistry for IDH1 R132H can be used to exclude the most frequent (about 90% of supratentorial IDH-mutant gliomas) mutation and this strategy has been found to be adequate for patients aged 55 and older since the probability of finding an alternative mutation in this setting is less than 1% [38]. However, no history suggesting a previous lower grade glioma should be present; otherwise, sequencing is warranted. To evaluate potential *IDH1*/*IDH2* mutations by sequencing, multiple assay types, including direct sequencing, can be used since the relevant mutations are restricted to exon 4 of *IDH1* and *IDH2*. Evaluation of *MGMT* promoter methylation will usually be indicated and possibly additional molecular profiling according to the local clinical practice, but this is not necessary for the diagnostic assessment.

If the morphological features of glioblastoma are lacking, but this diagnosis is suspected, for instance, if histology would be consistent with an IDH-wildtype lower grade astrocytic glioma in an older patient, the WHO 2021 classification provides for a molecular diagnosis of glioblastoma. To perform this diagnosis, a consistent DNA methylation profile can be detected or at least one of the following three markers should be present: chromosome 7 gain plus chromosome 10 loss and/or *TERT* promoter mutation and/or *EGFR* amplification. This choice is justified by the relative specificity of these markers in the right context and the similar outcome of patients diagnosed by these molecular criteria compared to those diagnosed according to morphological features [39–41]. In this setting, DNA methylation profiling would provide the diagnostic classification as an IDH-wildtype glioblastoma and would show the potential chromosome 7/10 alterations and/or *EGFR* amplification if present. If DNA methylation profiling is not available, an extensive DNA NGS panel can possibly demonstrate all these molecular markers. If a comprehensive assay with these characteristics is not available, chromosome 7 + /10 − and *EGFR* amplification can be detected by FISH or other suitable assays like MLPA, while *TERT* promoter status can be evaluated by sequencing assays.

Concerning the spectrum of IDH-mutant gliomas, diagnosis of an IDH-mutant astrocytoma requires histopathological findings consistent with an infiltrating diffuse glioma with an *IDH1*/*IDH2* mutation and ATRX loss/mutation or exclusion of 1p/19q codeletion. Conversely, IDH-mutant and 1p/19q codeleted oligodendroglioma requires the presence of whole-arm combined 1p/19 codeletion. Alternatively, diagnosis can be based on the detection of the corresponding methylation class. To ascertain ATRX status, immunohistochemistry can be used paying attention to potential artifacts due to necrosis and/or the presence of intermixed positive non-neoplastic reactive astrocytes. ATRX status can also be assessed by sequencing to detect loss of function mutation. *TP53*/p53 evaluation can also be helpful since it is frequently present in IDH-mutant astrocytomas resulting in diffuse positivity or, rarely, completely negative tumor cells [42, 43].

For diagnosing IDH-mutant, 1p/19q-codeleted tumors, a critical hallmark is the presence of whole-arm 1p/19q codeletion which can be investigated by multiple assays and FISH is commonly used [44]. However, due to the limited targeting of single loci by the FISH probes, false positive results are possible, especially in presence of tumors with complex karyotypes [45, 46]. In many cases, this occurrence is due to presence of partial deletions which are detected by FISH but are not biologically or diagnostically relevant. Since false positive FISH assessments of 1p/19q codeletion are frequently observed in cases for which 1p/19q status evaluation would not have been warranted (e.g., IDH-wildtype glioblastoma) [47], an appropriate and reasoned use of diagnostic tests can help avoid diagnostic pitfalls. If necessary, further testing (e.g., DNA methylation profiling, CNV profiling,…) is needed to conclusively define chromosomal status. Finally, it should be noted that *TERT* promoter mutations are frequently present in oligodendroglioma similarly to IDH-wildtype glioblastoma; this finding further highlights the importance of interpreting each molecular marker within the whole histopathological and clinical context.

Concerning the grading of IDH-mutant diffuse gliomas, morphological features play a critical role, but in the 2021 WHO classification, evaluation of *CDKN2A/B* status has been added as a grading criterion for IDH-mutant astrocytomas: in presence of homozygous *CDKN2A/B* deletion, grade 4 shall be assigned due to the association with an unfavorable outcome [48, 49]. *CDKN2A/B* status can be evaluated by visual inspection of the CNV plot gathered by DNA methylation profiling, through DNA NGS and by FISH, although a conclusive cut-off value has not been determined yet [50–52].

### High-grade astrocytoma with piloid features

High-grade astrocytoma with piloid feature (HGAP) is a novel tumor type recognized by the 2021 WHO classification of CNS tumors. This neoplasm has been mainly identified by its specific DNA methylation profile and is molecularly characterized by alterations in the MAPK pathway genes, homozygous deletion of *CDKN2A/B* and *ATRX* mutations.

HGAP most frequently occurs in adult patients and within the posterior fossa. Histopathological features of this tumor are markedly heterogenous and can include a glioblastoma or pleomorphic xanthoastrocytoma-like morphology, piloid features, and fibrillary aggregates (Rosenthal fibers or eosinophilic granular bodies). Vascular hypertrophy or proliferation is present in most cases, while necrosis is rarer [7]. Of note, due to this histopathological heterogeneity and overlap with other tumor types, histopathological findings do not allow a conclusive HGAP diagnosis by themselves, but a consistent DNA methylation profiling is required according to the 2021 WHO classification [2].

### Pediatric H3-altered diffuse gliomas

Pediatric high-grade gliomas with H3 alterations are another example of the importance of histopathological-molecular integration for reaching a correct diagnostic assessment. Two main glioma subtypes harboring H3 alterations are currently recognized: diffuse midline glioma, H3 K27-altered and diffuse hemispheric glioma, H3 G34-mutant.

The first one is typically diagnosed in children, although it can also occur in adults; in children, it usually arises in the brainstem/pons or with a bithalamic presentation, while in adults, it usually has a spinal or monothalamic localization [53]. Compared to the 2016 WHO classification of CNS tumors, multiple molecular subtypes of this entity have been defined with a common marker represented by loss of H3 K27 trimethylation which can be assessed by immunohistochemistry. One of the following molecular alterations is usually also present in combination: (i) a H3 K27M or, very rarely, K27I mutation in H3 coding genes (i.e., H3.3, H3.1 or, very rarely, H3.2 with clinical and prognostic correlations); (ii) *EGFR* mutation or amplification; (iii) EZHIP overexpression which characterizes the most rare subtype. H3 K27 mutations can be assessed by DNA sequencing, but immunohistochemistry is available for the H3 K27M mutation, potentially allowing to achieve this very specific diagnosis with minimal material consumption which is an important advantage considering that the location of these tumors can hamper their surgical sampling. *EGFR* alterations can be assessed by DNA NGS or by DNA sequencing for mutational analysis and FISH for amplification detection. Finally, EZHIP overexpression is commonly detected by immunohistochemistry [54]. Concerning the prognostic implications of this stratification, patients with H3.1 and H3.2 mutations have shown a longer survival than H3.3 mutant, although the overall outcome of this neoplasm is very dire [55].

Of note, loss of H3 K27 trimethylation is also a common feature of ependymoma, posterior fossa group A and H3 K27M mutations have been reported in multiple other tumor type; thus, correlation with histopathological features is critical [56, 57].

Diffuse hemispheric glioma, H3 G34-mutant is a high-grade tumor usually occurring in adolescents and young adults. Histopathological findings usually resemble a glioblastoma with high mitotic activity, microvascular proliferations and necrosis, but features suggesting an embryonal tumor can also be present. Diagnosis requires the demonstration of a H3 (H3.3) G34R (more than 90% of cases) or G34V mutation which can be detected by DNA sequencing or by immunohistochemistry since antibodies are available for the mutated proteins, but it should be noted that false negative results are possible. Prognosis is poor with a median overall survival of about 17 months [58].

Alternatively, diagnosis by DNA methylation profiling is also possible for both H3 K27-altered and H3 G34-mutant gliomas.

### Infant-type hemispheric glioma

Infant-type hemispheric glioma (IHG) is a high-grade astrocytoma frequently characterized by fusions of receptor tyrosine kinase genes including *ALK*, *ROS1*, *MET*, and *NTRK1*/*NTRK2*/*NTRK3*. IHG usually occur within the first age of life and are frequently diagnosed as a large neoplasm involving a cerebral hemisphere. Histopathological findings frequently resemble a glioblastoma, although a certain heterogeneity has been reported [59, 60]. Concerning the molecular profiling of these tumors, IHG is characterized by a specific DNA methylation profile and evaluation of potential gene fusions through RNA NGS or other assays is critical since the characteristic oncogenic gene fusions can frequently be therapeutically targeted and could also have prognostic implications [60].

### Ependymomas

**Fig. 3:**
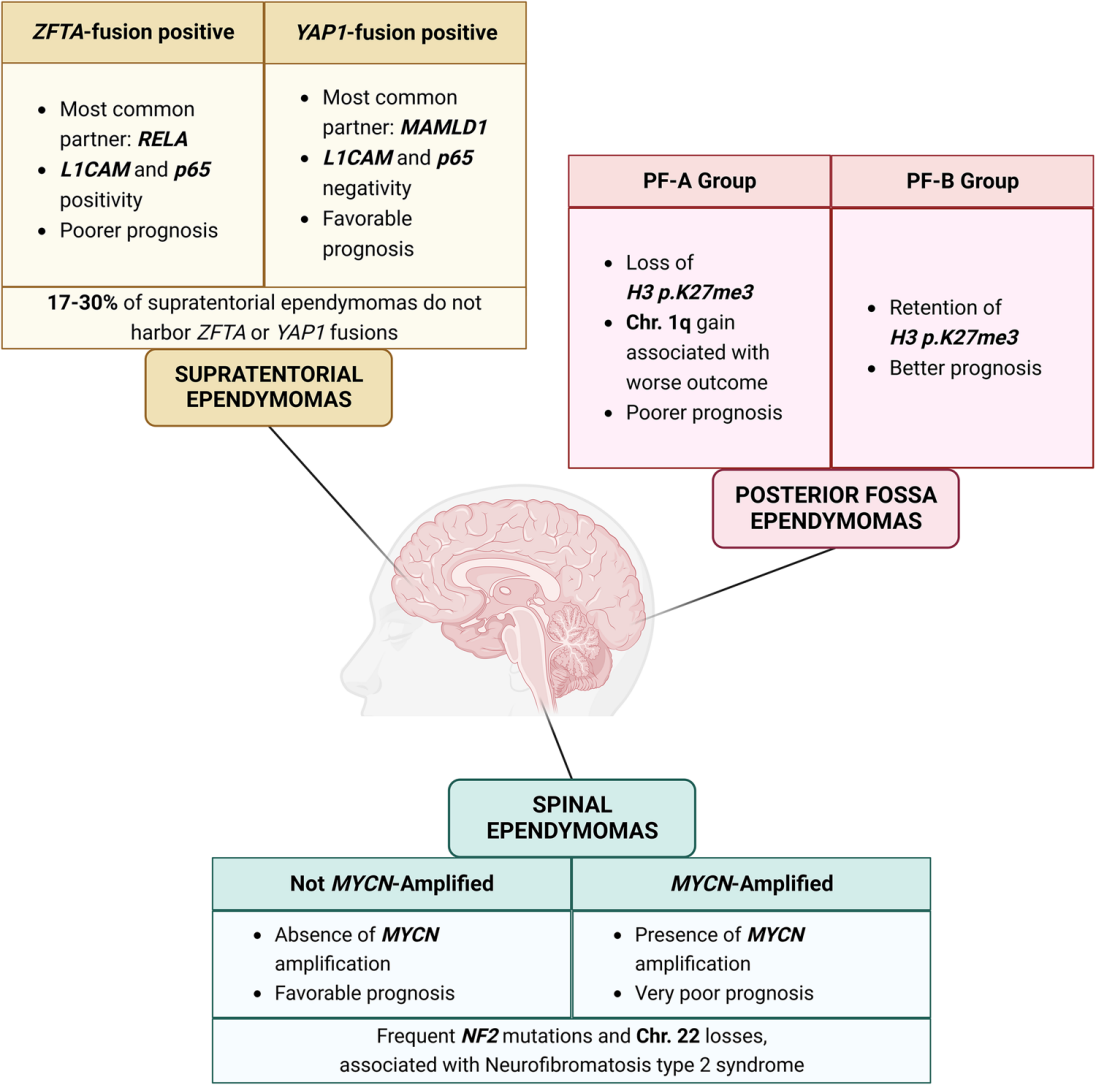
Overview of ependymoma molecular types according to the latest WHO 2021 classification of central nervous system tumors showing the relevant molecular hallmarks based on tumor site

Molecular subgrouping of ependymomas was already envisaged by the 2016 WHO classification of CNS tumors, but it has been significantly implemented by the latest classification (Fig. 3). Within supratentorial ependymomas, two specific, molecularly defined types have been added, based on the presence of *ZFTA* or *YAP1* fusions. Supratentorial ependymomas, *ZFTA* fusion-positive include the previous group of *RELA* fusion ependymomas since this is the most frequent partner of the ZFTA gene. Ependymomas with *ZFTA*-fusion can occur both in children and adults, and are associated with a poorer outcome, although recent prospective data shows a more favorable outcome compared to previous retrospective studies [61]. *CDKN2A/B* homozygous deletion has been found to be associated with dismal prognosis [62]. Supratentorial ependymomas, *YAP1* fusion-positive are rare, usually occurring in young children and *MAMLD1* is the most frequent *YAP1* gene partner [63]. RNA NGS can be used to investigate the presence of these gene fusions; other assays, like RT-PCR or FISH, can be used as well, but could not allow to identify the involved gene partner. DNA methylation profiling can also be used to distinguish the different molecular subgroups. Of note, up to 30% of supratentorial ependymomas do not harbor a *ZFTA* or *YAP1* fusion: a careful exclusion of other potential diagnoses should be performed in these cases.

Among posterior fossa ependymomas, tumor epigenetic profiles play a critical role and distinguish two main subgroups, group A (PFA) and group B (PFB). PFA is characterized by loss of H3 K27 trimethylation which can be also assessed by immunohistochemistry, while in PFB H3 K27 trimethylation is retained. However, it should be noted that, as previously discussed, H3 K27 trimethylation is also lost in midline H3-altered diffuse gliomas, while presence of H3 K27 trimethylation is unspecific since it can be observed both in neoplastic and non-neoplastic tissues. DNA methylation profiling can reliably distinguish PFA and PFB ependymomas, and also provide a prognostically relevant subgrouping [64]. Concerning molecular prognostic markers, chromosome 1q gain has been demonstrated to be associated with poorer prognosis [65].

Finally, within spinal ependymomas, a novel tumor type characterized by *MYCN* amplification has been introduced with the 2021 classification. This neoplasm is characterized by aggressive histopathological features and a poor outcome [66]. *MYCN* amplification can be detected by multiple assays including FISH and DNA methylation profiling which allows both to identify the specific methylation class of this tumor and the *MYCN* amplification in the derived CNV plot [67].

### Embryonal tumors

Within the 2021 classification of CNS tumors, two novel embryonal tumors have been added: CNS neuroblastoma, *FOXR2*-activated and CNS tumor with *BCOR* internal tandem duplication.

CNS neuroblastoma, *FOXR2*-activated is a rare neoplasm, usually occurring in children and frequently located in the cerebral hemispheres [68]. The histopathological spectrum varies from an undifferentiated, neuroblastic appearance to variable signs of neuronal differentiation with ganglion cells and a neuropil-rich stroma. Molecularly, this tumor is characterized by *FOXR2* rearrangements leading to its activation which are frequently associated with chromosome 1q gain. DNA methylation profiling can recognize this tumor type, while RNA NGS can be used to detect the *FOXR2* rearrangement [69]. Recently, an integrated diagnostic algorithm has been proposed for the diagnostic work-up of suspected CNS neuroblastomas, *FOXR2*-activated [70].

CNS tumor with *BCOR* internal tandem duplication (ITD) is also a pediatric embryonal neoplasm usually occurring in a cerebral or cerebellar hemisphere. The molecular hallmark of this tumor, *BCOR* ITD, is shared by multiple extra-CNS neoplasms leading to a debate regarding the neuroepithelial or mesenchymal nature of this tumor. Histopathological features are variable and can include ependymoma-like perivascular pseudorosettes as well as pseudopalisading necrosis, leading to multiple potential differential diagnoses. RNA and DNA NGS can allow to detect the *BCOR* ITD and exclude other molecular traits (including other types of *BCOR* alterations) which can be observed in other tumor types. CNS tumors with *BCOR* ITDs also harbor a specific DNA methylation profile [4].

### Meningiomas

As a last example, meningiomas are the most common primary brain tumor and thus a frequent diagnosis which can be encountered also outside of the neuropathology practice. Meningiomas are a heterogenous group of tumors with favorable outcomes in most cases. Diagnosis was based on histopathological findings only until the latest 2021 WHO classification, while now it encompasses both histopathological and molecular characteristics. Compared to the 2016 classification, a rhaboid or papillary histotype is no longer considered as a grading criterion by themselves, while presence of *TERT* promoter mutation and/or *CDKN2A/B* homozygous deletion has been added as independent criteria to assign grade 3 since meningiomas with these alterations display a significantly poorer prognosis independently of the histological features [71–73]. As seen for glioblastoma, *TERT* promoter mutations can be investigated by DNA sequencing, while *CDKN2A/B* status can be assessed by multiple tools including DNA NGS and FISH. Moreover, the use of immunohistochemical surrogates for *CDKN2A/B* assessment has been proposed, but available data is still limited [74]. DNA methylation profiling is also an effective prognostic tool, capable of providing significant outcome stratification [75, 76]. Of note, clear guidelines regarding which histologically grade 1 or 2 meningiomas should be submitted to molecular profiling are still missing, but integrated algorithms are being proposed to optimize the overall diagnostic workflow of meningiomas [77].

## Conclusion

Molecular neuropathology is based on a complex and rapidly evolving boxset of tools which have revolutionized our knowledge about CNS tumors and have reshaped the diagnostic workflow of these neoplasms. As shown by the provided examples, an effective and appropriate use of these instruments is contributory or even mandatory for reliably diagnosis of multiple tumor types but requires a multidisciplinary expertise in both diagnostic neuropathology and molecular pathology which should now be included in training programs. Hopefully, the diagnostic refinement enabled by these tools will also translate into clinical benefits and effective novel treatments for these neoplasms.
